# An Acetyl-CoA-Gated Metabolic Checkpoint Links Precursor Supply to Cordycepin Biosynthesis in *Cordyceps militaris*

**DOI:** 10.3390/cimb48040370

**Published:** 2026-04-02

**Authors:** Hucheng Zhang, Dongqing Yang, Guowei Yang, Shuai Luo, Peng Lu, Guoli Xie, Yapeng Song, Jun Yang, Linying Liu, Haitao Fan, Wenyan Lao, Frank Vriesekoop

**Affiliations:** 1 Bioengineering College, Beijing Polytechnic University, Beijing 100176, China; zhanghchbj@163.com (H.Z.); yangdongqing@bpu.edu.cn (D.Y.); yguow@163.com (G.Y.); luoshuai1985@sina.com (S.L.); lupeng531@163.com (P.L.); xieguoli1002@sina.com (G.X.); 103251@bpu.edu.cn (Y.S.); rainman@sina.com (J.Y.); llyspo@163.com (L.L.); 13587896605@163.com (H.F.); 2 School of Biochemical Engineering, Beijing Union University, Beijing 100023, China; 3 Harper Food Innovation, Harper Adams University, Newport TF10 8NB, UK

**Keywords:** cordycepin, *Cordyceps militaris*, proteomics, metabolic pathways, biosynthesis, secondary metabolites

## Abstract

The growth–specialized metabolism trade-off limits fungal natural product production. Here, we investigated cordycepin overproduction in *Cordyceps militaris* high-yield GYS60 and low-yield GYS80 via comparative proteomics, Post-Translational Modification (PTM) mapping, and metabolomics. We identified an acetyl-CoA-gated checkpoint centered on O-methyltransferase CCM_06472, whose activity is modulated by Lys123 acetylation and Ser34 phosphorylation in a manner consistent with activation and inhibition. GYS60 hyperactivates the tricarboxylic acid (TCA) cycle and β-oxidation to generate a 4.1-fold acetyl-CoA surplus, 62% of which is channeled into cordycepin synthesis. A single K123Q acetylation-mimetic mutation boosted cordycepin titers by >4-fold in wild-type strains. This acetyl-CoA checkpoint reveals PTM-gated flux allocation as a key regulatory mechanism, providing a minimal-intervention strategy for engineering fungal cell factories.

## 1. Introduction

The efficient microbial production of high-value natural products is fundamentally constrained by a pervasive biological trade-off, the competition for finite metabolic resources between essential cellular processes and the biosynthesis of specialized compounds [1]. This competition establishes a yield ceiling that synthetic biology and metabolic engineering strive to overcome [2]. For industrial strain development, simply boosting pathway flux often fails, because cells retain innate regulatory programs that prioritize survival over high-level production of secondary metabolites.

Fungi represent exceptional cell factories for pharmaceuticals, nutraceuticals, and agrochemicals, yet their complex and cryptic regulatory networks make yield improvement exceptionally challenging [3,4]. Traditional strategies, such as enzyme overexpression, promoter engineering, and fermentation optimization, increase biosynthetic capacity but frequently encounter diminishing returns [3,4]. A major limitation is that such approaches often neglect the dynamic metabolic flux allocation, the cellular decision-making process that directs precursors from primary to secondary metabolism [5].

Acetyl-CoA is a central metabolic hub that fuels both the TCA cycle and secondary metabolite biosynthesis [6,7]. Recent evidence highlights post-translational modifications (PTMs) [8], particularly lysine acetylation and phosphorylation, as dynamic regulators of metabolic flux in response to nutrient availability [9,10]. These PTM events enable cells to sense metabolic state and rewire enzyme activity without altering transcription or translation, making them ideal candidates for governing flux commitment.

**Figure 1:**
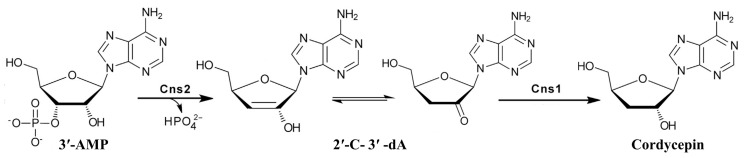
Simplified biosynthetic pathway of cordycepin in *C. militaris*. Note: The pathway proceeds from 3′-AMP via sequential catalysis by Cns2 (phosphohydrolase) and Cns1 (oxidoreductase/dehydrogenase), generating 2′-C-3′-dA as an intermediate before forming cordycepin. Both enzymes are indispensable for cordycepin biosynthesis.

In *Cordyceps militaris*, the core cordycepin biosynthetic pathway is controlled by the *cns1–2* gene cluster [11,12,13]. *Cns1* encodes an oxidoreductase/dehydrogenase, and *cns2* encodes a metal-dependent phosphohydrolase; both are essential for cordycepin formation. This pathway branches from purine metabolism and uses 3′-adenosine monophosphate (3′-AMP) as a central precursor, and a simplified biosynthetic pathway is illustrated in Figure 1. However, the regulatory mechanisms underlying strain-specific yield differences remain unclear [14,15,16], creating a critical barrier to rational engineering of high-yield strains. *C. militaris* produces cordycepin, a nucleoside analog with potent pharmacological activities [17,18,19,20], but wild-type yields are economically limited. Transcriptional profiling provides only partial insight into yield divergence [16,21], indicating that post-transcriptional and PTM-dependent regulation dominate the high-yield phenotype.

We hypothesized that high-yield fungi evolve PTM-encoded metabolic checkpoints to rewire resource allocation by gating key enzyme activity in response to precursor availability. Using the GYS60/GYS80 system [14], we integrated comparative proteomics, PTM mapping, and metabolomics to identify regulatory nodes, metabolic drivers, and flux outcomes. Here, we report an acetyl-CoA-gated metabolic checkpoint centered on O-methyltransferase CCM_06472, whose activity was controlled by antagonistic acetylation and phosphorylation. This checkpoint links acetyl-CoA abundance to cordycepin flux, and a single PTM-mimetic mutation boosts yield by over 4-fold. Our study establishes PTM-gated flux allocation as a unifying regulatory principle and provides a streamlined blueprint for fungal cell factory engineering.

## 2. Materials and Methods

### 2.1. Strains, Cultivation, Sample Preparation, and Protein Extraction

The high-cordycepin-yield strain GYS60, wild-type (WT), and low-yield strain GYS80 (preserved in the Culture Collection of Beijing Polytechnic University) were cultivated as described previously [14,16]. Mycelia were harvested by vacuum filtration, washed with ice-cold water, flash-frozen in liquid nitrogen, and stored at −80 °C. A subset of fresh mycelia was lyophilized for initial cordycepin quantification via HPLC-UV to verify the yield differences prior to omics analysis [14].

**Figure 2:**
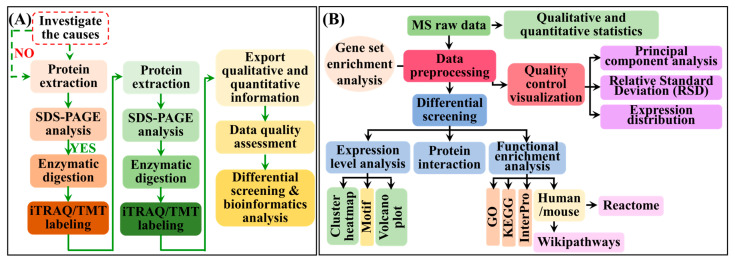
Integrated omics workflow for dissecting cordycepin biosynthesis regulation in *C. militaris*. (**A**) Experimental workflow for proteomic and PTM analyses. (**B**) Bioinformatics pipeline for data integration.

Proteins were extracted from the ground mycelia and quantified using the BCA assay. For proteomic analysis, 100 μg of protein per sample was processed using the filter-aided sample preparation (FASP) method. Briefly, proteins were reduced, alkylated, and digested with trypsin (1:50 *w*/*w*) overnight at 37 °C. The resulting peptides were desalted using C18 StageTips, dried under vacuum, and stored at −20 °C until further use. The complete proteomic workflow is illustrated in Figure 2A, with the bioinformatics pipeline shown in Figure 2B.

### 2.2. LC-MS/MS-Based Proteomics and PTM Profiling

#### 2.2.1. PTM Enrichment and Tandem Mass Tag (TMT) Labeling

Two key PTMs (phosphorylation and acetylation) were enriched from the desalted peptides. For phosphorylation enrichment, desalted peptides (100 μg) were incubated with TiO_2_ magnetic beads in a loading buffer (80% ACN and 5% TFA) for 30 min at room temperature. After washing, the phosphorylated peptides were eluted with 5% ammonia water, acidified, desalted, and dried. For acetylation enrichment, peptides (100 μg) were incubated overnight at 4 °C with anti-acetyl-lysine antibody-conjugated agarose beads (Cell Signaling Technology, #9681, Danvers, MA, USA) in IP buffer. After washing, the bound peptides were eluted with 0.1% trifluoroacetic acid (TFA), desalted, and dried. All PTM-enriched peptide samples were prepared in biological triplicates before LC-MS/MS analysis.

TMT16-plex labeling was performed according to the manufacturer’s protocol (Thermo Fisher Scientific, Waltham, MA, USA). Briefly, peptides from biological quadruplicates of each strain (GYS60, GYS80, and WT) were resuspended in 100 mM triethylammonium bicarbonate (TEAB), labeled with the respective TMT reagents for 1 h, and the reaction was quenched with hydroxylamine. The labeled peptides from individual strains were pooled to minimize batch effects. The pooled samples were then fractionated by high-pH reversed-phase HPLC using an Agilent 1260 system (XBridge BEH C18 column, Santa Clara, CA, USA) with a linear acetonitrile gradient (5–35% B in 45 min; mobile phase A: 10 mM ammonium formate, pH 10; B: 90% ACN, 10 mM ammonium formate, pH 10). Fractions were collected at 1 min intervals, concatenated into 12 fractions, dried, and reconstituted for LC-MS/MS analysis.

#### 2.2.2. LC-MS/MS Analysis

LC-MS/MS analysis was performed using a Q Exactive HF-X mass spectrometer (Thermo Fisher Scientific, Waltham, MA, USA) coupled to an EASY-nLC 1200 system (Figure 2A). Peptides were separated on a 75 μm × 25 cm C18 column with a 75 min gradient of 5–28% acetonitrile (in 0.1% formic acid) at 300 nL/min. Mass spectrometry data were acquired in the data-dependent acquisition (DDA) mode. Full MS scans (350–1800 *m*/*z*) were acquired at 60,000 resolution, followed by higher-energy collisional dissociation (HCD) fragmentation (normalized collision energy, NCE 32%) of the top 20 most intense precursors.

#### 2.2.3. Data Processing and Quantification

Raw files were processed using Proteome Discoverer (v2.5) against a custom *C. militaris* database (UniProt, EMBL-EBI, Hinxton, UK supplemented with 21 known cordycepin biosynthetic genes). Search parameters included trypsin digestion (up to 2 missed cleavages), static modifications (TMT16plex on N-terminus and Lys, carbamidomethyl on Cys), and dynamic modifications (Met oxidation, Ser/Thr/Tyr phosphorylation, Lys/N-terminus acetylation). Peptide and protein identifications were filtered at a 1% false discovery rate (FDR) using a target–decoy strategy. TMT reporter ion intensities were median-normalized, and only proteins quantified with a coefficient of variation (CV) < 20% across biological replicates were retained for further analysis.

### 2.3. Metabolomic and Isotope-Tracer Flux Analysis

#### 2.3.1. Non-Targeted Metabolomic Analysis

For metabolite extraction, frozen mycelia (50 mg) were homogenized in ice-cold methanol: water: chloroform (5:2:2, *v*/*v*/*v*). After centrifugation, the polar (aqueous) phase was collected, dried, and reconstituted in 0.1% formic acid for LC-MS. Non-targeted metabolomics was performed using a Waters ACQUITY UPLC I-Class (Waters, MA, USA) system coupled to a Xevo G2-XS Q-TOF (Waters, MA, USA) mass spectrometer. Chromatographic separation was achieved using an HSS T3 column (2.1 mm × 100 mm, 1.8 μm) with a water/acetonitrile gradient (both with 0.1% formic acid) over 15 min. MS data were acquired in the ESI+ mode (50–1200 *m*/*z*). Data processing, including peak alignment and annotation, was performed using the Progenesis QI software (v2.3).

#### 2.3.2. Targeted Metabolomic Analysis

Targeted quantification of cordycepin and its biosynthetic precursors (adenine, adenosine, and AMP) was performed using an Agilent 1290 HPLC-UV (Agilent, Santa Clara, CA, USA) system equipped with a ZORBAX SB-C18 (Agilent, Santa Clara, CA, USA) column (4.6 mm × 250 mm, 5 μm). Chromatographic separation was achieved with a gradient of methanol and water (*v*/*v*) at a flow rate of 1.0 mL/min, and detection was conducted at 260 nm. Concentrations were determined using external standard curves with *R*^2^ ≥ 0.998.

#### 2.3.3. Isotope-Tracer Flux Analysis

To quantify acetyl-CoA flux partitioning between cordycepin biosynthesis and competing pathways, ^13^C-glucose (99% ^13^C6, Sigma-Aldrich, St. Louis, MO, USA) was added to liquid cultures at 20 g/L when mycelial dry weight reached 1.0 g/L [22]. Cultures were harvested at 24 h post-labeling to ensure labeled metabolite accumulation. Targeted quantification of ^13^C-labeled metabolites was performed via UPLC-MS/MS (Waters Xevo TQ-S, Milford, MA, USA) in selected reaction monitoring (SRM) mode. Due to free-acetyl-CoA instability, its labeled pool was quantified via the stable surrogate acetylcarnitine (*m*/*z* 204.1 → 85.0), with labeled cordycepin (*m*/*z* 252.1 → 135.0) and riboflavin (*m*/*z* 377.1 → 243.0) quantified directly, all ion pairs validated previously [22]. Fractional labeling (FL) was calculated as labeled/total peak area using MassLynx v4.2. Acetyl-CoA flux allocation was estimated using (Equation (1)):

(1) $${Flux}_{allocation}\left(\%\right)=\frac{{FL}_{target}\times M_{target}\times Stoichiometry}{\Sigma \left({FL}_{pathway}\times M_{pathway}\times Stoichiometry\right)}\times 100$$

where FL_target_ = fractional labeling of cordycepin/riboflavin; M_target_ = molar concentration of products; Stoichiometry = 1 mole acetyl-CoA per mole cordycepin, 4 moles per mole riboflavin.

### 2.4. Enzyme Activity Assays, Western Blot Validation and Molecular Dynamics (MD) Simulation

#### 2.4.1. Enzyme Activity Assays

To assess the functional correlation between protein abundance and activity, we measured the activities of O-methyltransferase (CCM_06472). CCM_06472 activity was quantified by detecting 3′-O-methyl-AMP formation, with 3′-AMP as substrate and S-adenosylmethionine (SAM) as methyl donor. Reactions containing crude protein extracts were incubated at 30 °C for 1 h, terminated with trifluoroacetic acid (TFA), and then analyzed by UPLC-MS/MS. Specific activity was calculated from the difference in product yields with and without preincubation with in-house anti-CCM_06472 polyclonal antibody at 1:100 dilution. Activity was expressed as pmol 3′-O-methyl-AMP formed per hour per milligram of protein.

#### 2.4.2. Western Blot Validation

For Western blot validation, 30 μg of total protein per lane was separated by 12% SDS-PAGE and transferred to PVDF membranes. Membranes were probed with primary antibodies at 4 °C overnight, followed by HRP-conjugated secondary antibodies at 25 °C for 1 h. Signals were detected via ECL. The custom polyclonal anti-CCM_06472 antibody was generated using a recombinant N-terminal antigen (residues 1–30) with *E. coli* BL21 (DE3) expression and Ni-NTA purification to ≥95% homogeneity, and further purified via protein A/G agarose beads to a final concentration of 1.2 mg/mL. Specificity was validated by peptide blocking (antigen pre-incubation abolished target detection), exclusive recognition of ~45 kDa CCM_06472 with no non-specific bands, and specific detection in OE-CCM_06472 strains and absence in mock controls. WB conditions included anti-CCM_06472 at 1:1000, HRP-conjugated goat anti-rabbit IgG from Cusabio (Cat. CSB-PA489724, Wuhan, China) at 1:10,000, and GAPDH internal control from Cusabio (Cat. CSB-MA000071M1m) at 1:5000.

#### 2.4.3. Molecular Dynamics (MD) Simulation

MD simulations of CCM_06472 used the AlphaFold2-predicted structure [23,24] as the initial model. Run in GROMACS 2023.2 with the AMBER99SB-ILDN force field [25], models were solvated (TIP3P, 10 Å buffer, 0.15 M NaCl), minimized, equilibrated (300 K/1 bar), and simulated for 100 ns (n = 3). Root Mean Square Fluctuation (RMSF) was calculated via GROMACS; structural visualizations (key residue sticks) via AlphaFold2. Ribbon colors indicate AlphaFold2 pLDDT confidence (dark blue >90, light blue 70–90, yellow 50–70, orange <50).

### 2.5. Data Integration, Statistical Analysis

Proteomics and metabolomics data integration and statistical analyses were performed as follows, with key workflows shown in Figure 2B. Pearson correlations between differentially expressed proteins (DEPs) and differentially abundant metabolites (DAMs), notably those in cordycepin biosynthesis, were computed using MetaboAnalyst 5.0. Significance was defined as |*r*| ≥ 0.7, and *p* < 0.05. Protein–metabolite networks were visualized using Cytoscape v3.10. DEPs (|log_2_FC|) ≥ 1.5, *p* < 0.05) and DAMs (|log_2_FC|) ≥ 1, *p* < 0.05) were identified using one-way ANOVA with Tukey’s test in GraphPad Prism 9.0. Gene Ontology (GO)/Kyoto Encyclopedia of Genes and Genomes (KEGG) enrichment of DEPs was analyzed using cluster Profiler 4.4.0 (R package), with significance set at *p* < 0.05 and *q* < 0.1.

All quantitative experiments were performed with four independent biological replicates unless stated otherwise. Data are shown as mean ± SD, with CV < 15% considered acceptable. Student’s *t*-test was used for two-group comparisons, and Pearson’s test for correlation analyses.

## 3. Results

### 3.1. Central Carbon Metabolic Reprogramming Drives the High-Yield Phenotype

**Figure 3:**
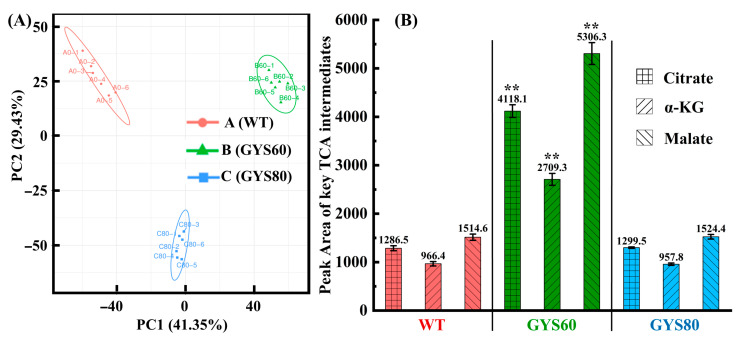
TCA cycle hyperactivation drives metabolic reprogramming for high cordycepin yield in GYS60. (**A**) Principal component analysis (PCA) score plot of untargeted metabolomics (n = 6). Groups: A = WT, B = GYS60, C = GYS80. PC1/PC2 explained >70% variance; Group B clustered distinctly. (**B**) Key TCA cycle intermediates (n = 6): Citrate, α-ketoglutarate, and malate in GYS60 were 3.2–3.5-fold higher than WT (** *p* < 0.01), with no significant changes in GYS80.

The high-yield phenotype of GYS60 was underpinned by a global reprogramming of central carbon metabolism. Untargeted metabolomics revealed a fundamental transformation of the metabolite profile in GYS60 compared to low-yield GYS80 and wild type (WT) (Figure 3A). The signature feature was not merely the accumulation of cordycepin precursors but a pronounced amplification of central carbon metabolism. Specifically, the concentrations of key TCA cycle intermediates, including citrate, α-ketoglutarate, and malate, increased by 3.2-fold, 2.8-fold, and 3.5-fold, respectively (*p* < 0.01, Figure 3B).

**Figure 4:**
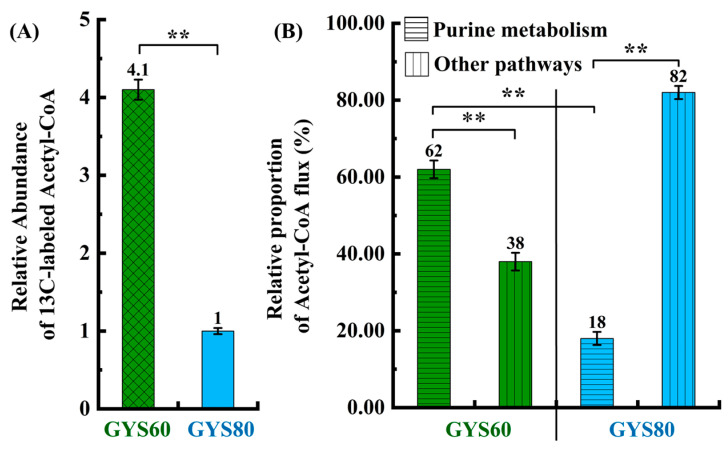
Acetyl-CoA surplus and selective flux allocation in GYS60 underpin cordycepin overproduction. (**A**) ^13^C-glucose tracing shows higher labeled acetyl-CoA in GYS60 vs. GYS80. (**B**) Distribution of acetyl-CoA metabolic flux between GYS60 and GYS80. ** *p* < 0.01.

Stable isotope tracing with ^13^C-glucose revealed a 4.1-fold higher abundance of labeled acetyl-CoA in GYS60 than in GYS80 (Figure 4A). Based on ^13^C-labeling data, stoichiometric correction and flux partitioning analysis revealed a stark difference in resource allocation. In GYS60, 62% of the additional acetyl-CoA was efficiently channeled into cordycepin biosynthesis (Figure 4B). In contrast, GYS80 diverted the majority of this flux (only 18% to cordycepin) into alternative pathways, notably riboflavin synthesis, resulting in a 3.2-fold higher riboflavin titer than that of GYS60. This demonstrates that GYS60 has a mechanism for efficient metabolic prioritization.

### 3.2. Proteomics and PTM Profiling Identify CCM_06472 as a Key Regulator

#### 3.2.1. Quality Assurance of Proteomic and PTM Datasets

**Figure 5:**
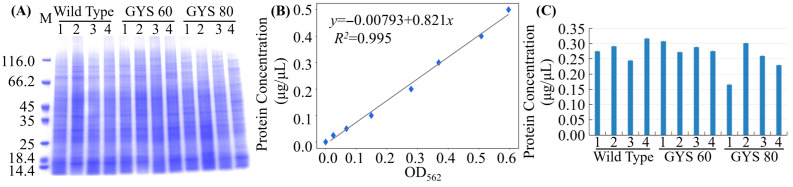
Quality control of proteomic samples for comparative proteomic analysis. (**A**) SDS-PAGE of extracts from WT, GYS60, and GYS80. (**B**) BCA standard curve for protein quantification. (**C**) OD_562_ values from (**B**) to normalize protein loads and reduce technical bias.

Conventional transcriptomics revealed limited differential expression (<2-fold) of core cordycepin biosynthetic genes between strains, suggesting post-transcriptional regulation [16]. Therefore, we conducted comprehensive comparative proteomics and post-translational modification (PTM) profiling with rigorous quality control to ensure analytical reliability (Figure 5). SDS-PAGE verified the integrity and uniformity of total protein extracts across WT, GYS60, and GYS80 strains (Figure 5A); a highly linear BCA standard curve (*R*^2^ = 0.995) enabled accurate protein quantification (Figure 5B); and OD_562_-based normalization of protein loads minimized technical bias in subsequent experiments (Figure 5C).

**Figure 6:**
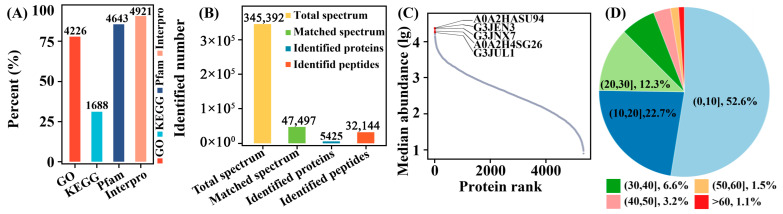
Proteomic coverage and quality metrics in *C. militaris* strains. (**A**) Functional annotation rates to map cordycepin biosynthetic enzymes. (**B**) Protein sequence coverage (<20% for most) with no strain bias. (**C**) Log-transformed protein abundance ranking; top 5 are glycolysis-related core metabolic proteins. (**D**) Unique peptides per protein (>80% have 2–10), confirming high-confidence identification.

High functional annotation rates (Figure 6A) and uniform protein sequence coverage (<20% for most, Figure 6B) validated pathway mapping and unbiased profiling. Log-transformed abundance ranking highlighted glycolysis-related core metabolic proteins as the most abundant (Figure 6C), with >80% of proteins supported by 2–10 unique peptides (Figure 6D), ensuring high-confidence identification.

**Figure 7:**
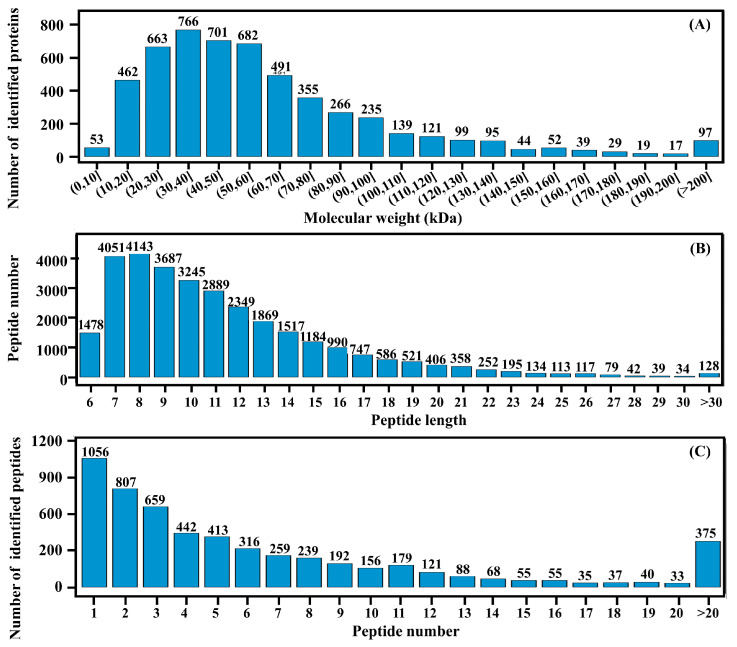
Proteomic quality control and coverage of *C. militaris* strains. (**A**) Protein molecular weight (10–100 kDa), covering regulatory kinases and TCA enzymes for cordycepin synthesis. (**B**) Peptide length (92% 7–20 aa, <8% missed cleavages) confirms robust FASP tryptic digestion. (**C**) High-confidence proteins after filtering (removal of contaminants, <2 unique peptides, FDR > 1%).

Protein molecular weight (10–100 kDa, Figure 7A) and peptide length (7–20 aa, <8% missed cleavages, Figure 7B) confirmed coverage of regulatory and metabolic enzymes, while strict filtering yielded high-confidence proteins (Figure 7C).

#### 3.2.2. Global PTM Landscapes Differentiate High- and Low-Yield Strains

**Figure 8:**
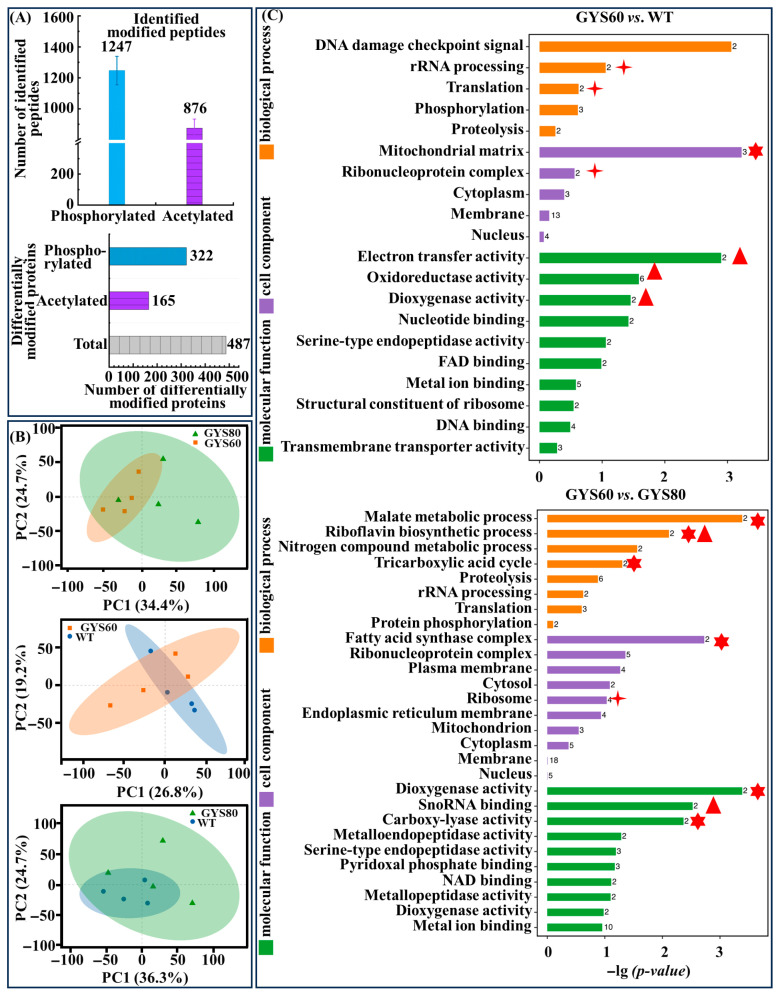
Global PTM landscape in high-yield GYS60. (**A**) Quantification of PTM events. A total of 1247 phosphorylated and 876 acetylated peptides were identified with 487 differentially modified proteins in GYS60. (**B**) PCA of PTM profiles. Distinct separation among GYS60, WT, and GYS80 confirms PTM patterns linked to cordycepin yield. (**C**) GO enrichment analysis. Top: GYS60 vs. WT; Bottom: GYS60 vs. GYS80. Red stars mark significantly enriched GO terms (*p* < 0.05): red multi-angle stars represent the TCA cycle, red triangles represent protein phosphorylation, and red crosses represent the oxidation–reduction process.

We identified 1247 phosphorylated and 876 acetylated peptides with high confidence (Figure 8A). Phosphoproteins exhibited a median sequence coverage of 19.3%, consistent with the global proteome coverage range (18–25%, Table 1), ruling out selection bias during enrichment.

Notably, 92.1% of the acetylated peptides mapped to lysine residues of metabolic enzymes (Figure 8A), including malate dehydrogenase (CCM_04892) and the key O-methyltransferase (CCM_06472) implicated in cordycepin biosynthesis (Figure 8A), directly linking PTM reprogramming to the target pathway. This confirmed that our enrichment strategy targeted core metabolic nodes governing the production phenotype. Strikingly, 487 proteins displayed significant PTM alterations between the strains (*p* < 0.05, Figure 8A). Principal component analysis (PCA) revealed that PTM profiles provided clearer separation between GYS60, GYS80, and WT than total protein abundance alone (Figure 8B), indicating that post-translational regulation is a key driver of phenotypic divergence. GO enrichment analysis linked differential PTMs to metabolic regulation (Figure 8C). GYS60 vs. WT enriched TCA cycle and protein phosphorylation, while GYS60 vs. GYS80 highlighted riboflavin biosynthesis (acetyl-CoA competing pathway) and fatty acid metabolism, tying PTMs to flux allocation.

#### 3.2.3. CCM_06472 Functions as a Core PTM-Gated Regulatory Node

**Figure 9:**
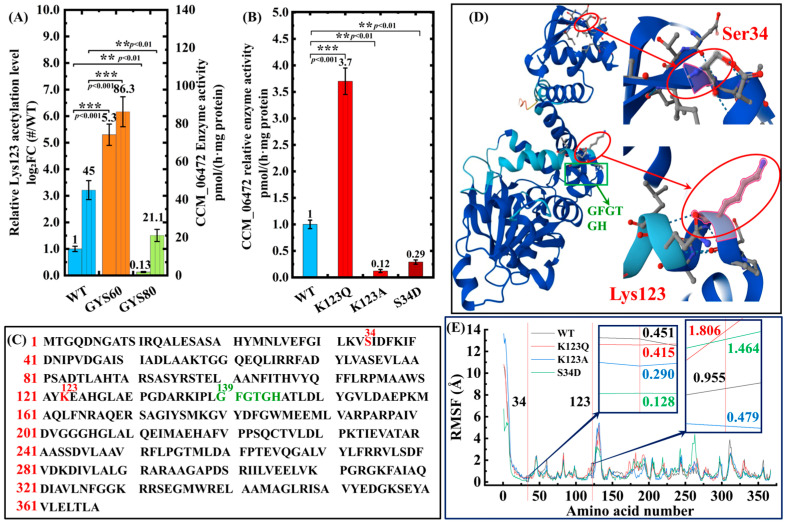
Functional–structural validation of CCM_06472 as a key regulatory node. (**A**) Correlation between Lys123 acetylation and CCM_06472 activity (*r* = 0.92, *p* < 0.001). (**B**) Mutagenesis assays. K123Q enhances enzyme activity 3.7-fold, K123A abolishes activity, and S34D inhibits enzyme activity by 71%. (**C**) Sequence alignment shows conserved Ser34, Lys123, and the SAM-binding motif. Red residues (S34, K123) mark identified PTMs. Green residues (GFGTGH) denote conserved residues. (**D**) AlphaFold2 structure showing Lys123 (flexible) and Ser34 (low-flexibility). Color coding confidence (dark blue: pLDDT > 90; blue: 70–90; yellow: 50–70). (**E**) RMSF profiles showing residue flexibility in WT and mutants.

A strong positive correlation between CCM_06472 Lys123 acetylation and methyltransferase activity (*r* = 0.92, *p* < 0.001) supports PTM as the dominant regulatory layer (Figure 9A). Lys123 acetylation increased 5.3-fold in GYS60 and dropped to 0.13-fold in GYS80, with enzyme activity following the same trend. Site-directed mutagenesis is consistent with a causal role of Lys123 acetylation in the PTM switch (Figure 9B). Acetylation-mimetic K123Q boosted activity 3.7-fold, while phosphomimetic S34D inhibited it by 71%, defining an acetylation-activation/phosphorylation-inhibition mechanism. Sequence alignment (Figure 9C) demonstrates that CCM_06472 retains the phosphorylation site Ser34, acetylation site Lys123, and the conserved SAM-binding motif (GFGTGH) across WT, GYS60, and GYS80 strains. Full-length sequence alignment further verifies that the amino acid sequence of CCM_06472 is identical across the three strains (Figure 9C), with no genetic mutations detected in the coding region.

AlphaFold2 modeling (Figure 9D) and 100 ns molecular dynamics (MD) simulations (Figure 9E) showed that in WT CCM_06472, Ser34 (phosphorylation site) localizes to a low-flexibility surface region (RMSF = 0.451 Å), while Lys123 (acetylation site) resides in a moderately flexible surface loop (RMSF = 0.955 Å). This distribution aligns with the consensus that functional PTM sites occupy low-to-moderately flexible surface regions (0.3–2.0 Å), avoiding highly flexible areas (>2.0 Å) that preclude stable enzyme recognition [23,24,25]. Structural comparison of WT with K123Q (acetylation-mimetic) and S34D (phosphomimetic) mutants revealed distinct conformational dynamics: the K123Q mutation significantly increased Lys123 flexibility by 0.851 Å (to 1.806 Å), adopting an extended, active conformation; the S34D mutation reduced Ser34 flexibility by 0.323 Å (to 0.128 Å), rigidifying the loop; and the acetylation-deficient K123A mutation decreased Lys123 flexibility by 0.476 Å (to 0.479 Å) (Figure 9E). This conformational divergence explains the differing PTM enzyme recognition efficiency across strains. WT flexibility enables robust modification, while mutations abrogate it, consistent with RMSF data (Figure 9E) and supporting CCM_06472’s PTM-gated regulation.

### 3.3. CCM_06472 Acts as an Acetyl-CoA-Responsive Metabolic Switch

Mining the comparative PTM network identified a previously uncharacterized O-methyltransferase (CCM_06472) as a pivotal node linking acetyl-CoA metabolism to cordycepin biosynthesis (Table 2). Although its protein abundance increased only moderately in GYS60 (2.94-fold vs. WT, *p* < 0.05; Table 3), the acetylation status of Lys123 diverged dramatically across strains (Figure 9A). The acetylation level at Lys123 strongly correlated with CCM_06472 methyltransferase activity (*r* = 0.92, *p* < 0.001), supporting PTM, rather than abundance, as a dominant regulatory layer for this enzyme.

Figure 9A couples Lys123 acetylation with CCM_06472 activity via dual Y-axes: the left denotes relative acetylation (WT = 1.0), and the right catalytic activity (3′-AMP → 3′-O-methyl-AMP, pmol/(h·mg protein)). Relative to WT, Lys123 acetylation increased 5.3-fold in GYS60 but decreased to 0.13-fold in GYS80. Activity paralleled this trend: 86.3 ± 7.9 (GYS60, 4.1-fold vs. GYS80’s 21.0 ± 3.2) and 45 ± 5.2 (WT) pmol/(h·mg protein). This tight correlation (*r* = 0.92, *p* < 0.001; Table 2) supports that Lys123 acetylation, not protein abundance, is tightly associated with CCM_06472 activity. In vitro assays further showed that Lys123 acetylation depends on acetyl-CoA concentration, supporting CCM_06472 as a metabolic responder to acetyl-CoA levels.

Site-directed mutagenesis is consistent with a causal role of Lys123 acetylation. Mimicking constitutive acetylation (K123Q) boosted enzyme activity 3.7-fold, whereas an acetylation-deficient mutant (K123A) nearly abolished the activity. Conversely, a phosphomimetic mutation (S34D) inhibited activity by 71% (Figure 9B). This defines a precise “acetylation-activation, phosphorylation-inhibition” molecular switch in CCM_06472 that regulates cordycepin biosynthetic flux.

Metabolic engineering of this switch, co-expressing a Lys123 acetyltransferase (CCM_04567) and knocking down Ser34 kinase (CCM_08910), increased GYS60’s cordycepin yield 2.3-fold, highlighting the biotechnological potential of PTM code decoding.

### 3.4. Acetyl-CoA Serves as a Metabolic Signal to Gate the PTM Switch

#### 3.4.1. Acetyl-CoA Activates CCM_06472 in a Dose-Dependent Manner

To define the upstream signal governing CCM_06472 PTM-dependent activation, we focused on acetyl-CoA, an integration node of central metabolism and PTM regulation. In vitro reconstitution assays were fitted for a four-parameter sigmoidal dose-dependent model (Equation (2)) to quantify acetyl-CoA-dependent activation of CCM_06472:

(2) $$v=Basal+\frac{V_{max}-Basal}{1+10^{(lg{EC}_{50}-\left[S]\right)*Hillslope}}$$

Equation (2): Sigmoidal dose–response model for acetyl-CoA-dependent CCM_06472 activation. v: Net CCM_06472 methyltransferase activity (pmol/(h·mg protein)). Basal: Basal enzyme activity (0 μM acetyl-CoA) = 15.2 pmol/(h·mg protein). Vmax: Maximal enzyme activity (saturated acetyl-CoA) = 119.0 pmol/(h·mg protein). EC50: Half-maximal activation concentration = 25.8 μM. [S]: Acetyl-CoA concentration (μM). HillSlope: Cooperativity coefficient = 2.3 (positive cooperativity).

**Figure 10:**
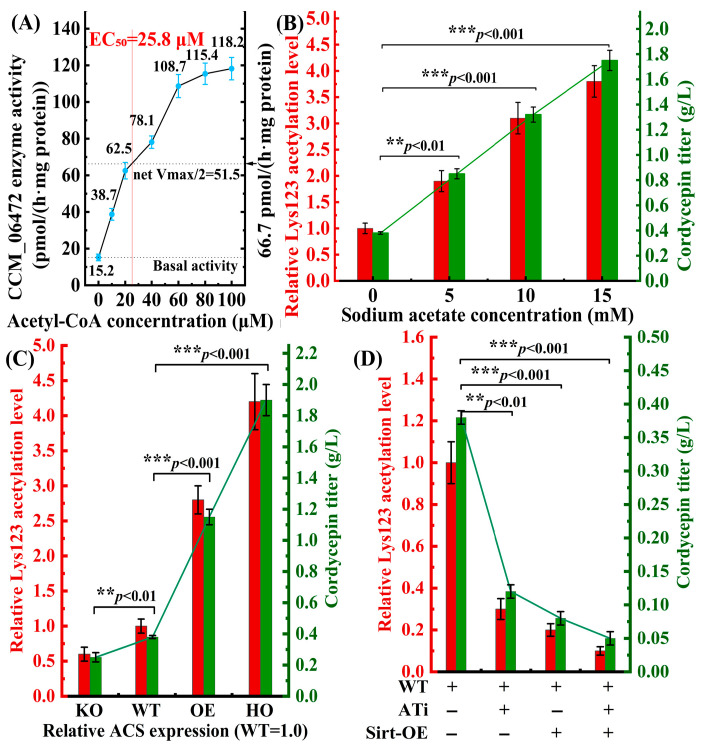
Acetyl-CoA-dependent acetylation of CCM_06472 Lys123 modulates cordycepin biosynthesis. (**A**) In vitro CCM_06472 activity exhibits a sigmoidal response to acetyl-CoA (EC_50_ = 25.8 μM). (**B**) Sodium acetate (0–15 mM) linearly induces Lys123 acetylation (3.8-fold) and cordycepin yield (*r* = 0.94, *p* < 0.001). (**C**) ACS overexpression enhances acetyl-CoA, Lys123 acetylation (4.2-fold), and cordycepin yield (1.90 g/L), while ACS knockdown reduces both by ≥40%. (**D**) Acetylation inhibition (ATi/Sirt-OE) reduces Lys123 acetylation (0.1–0.3-fold) and cordycepin yield (0.05–0.12 g/L).

Fitting of experimental data to Equation (2) supports acetyl-CoA as an activator of CCM_06472 in vitro, with the enzyme exhibiting positive cooperative dose dependence on acetyl-CoA (Figure 10A, *R*^2^ = 0.99, 95% CI for EC_50_: 23.1–28.5 μM). This EC_50_ corresponded to half the maximal net activity (net V_max_/2 = 51.5 pmol/(h·mg protein)), supporting acetyl-CoA as a dose-dependent trigger for CCM_06472. Further specificity validation showed that preincubation with anti-CCM_06472 antibody abrogated >90% of acetyl-CoA-induced activity, supporting that the response is specific to CCM_06472 and consistent with acetyl-CoA as a concentration-dependent regulatory signal for the enzyme in crude extract assays. To validate this in vitro regulation in live *C. militaris*, we manipulated intracellular acetyl-CoA pools via sodium acetate supplementation, direct conversion by acetyl-CoA synthetase [22].

#### 3.4.2. Chemical Modulation of Acetyl-CoA Recapitulates Enzyme Activation

We validated the in vitro findings in live *C. militaris* cells by manipulating acetyl-CoA pools. We supplemented WT strains with sodium acetate (0–15 mM), which converts to acetyl-CoA via acetyl-CoA synthetase, enabling analysis of acetyl-CoA’s impact on Lys123 acetylation and cordycepin yield (Figure 10B). IP-MS/MS quantified Lys123 acetylation, while HPLC-UV measured cordycepin yield. The results showed a strong correlation between sodium acetate concentration, Lys123 acetylation, and cordycepin production (Pearson *r* = 0.94, *R*^2^ = 0.89, *p* < 0.001). At 15 mM sodium acetate, Lys123 acetylation increased 3.8-fold versus untreated WT, with cordycepin yield reaching 1.75 g/L, comparable to GYS60’s yield (1.82 g/L, Table 4). This supports that elevated acetyl-CoA levels are associated with activation of the CCM_06472 PTM switch and drive high cordycepin production in WT strains.

#### 3.4.3. Genetic Manipulation of Acetyl-CoA Pool Confirms Regulatory Specificity

We genetically modulated acetyl-CoA levels by altering ACS (CCM_01567) expression (Figure 10C), generating four strains, including ACS-KO (RNAi knockdown, ~40% residual expression), WT (endogenous), ACS-OE (*gpdA* promoter, 2.0-fold vs. WT), and ACS-HO (tandem *gpdA* promoters, 3.5-fold vs. WT). Intracellular acetyl-CoA levels correlated with ACS expression, reflected in Lys123 acetylation and cordycepin yield: ACS-HO showed 4.2-fold higher Lys123 acetylation and 1.90 g/L cordycepin (4.3-fold over WT), while ACS-KO showed ≥40% reductions in both (0.23 g/L). This supports acetyl-CoA pool size as a determinant of PTM switch activation.

#### 3.4.4. Acetylation-Specific Intervention Supports Regulatory Roles

**Figure 11:**
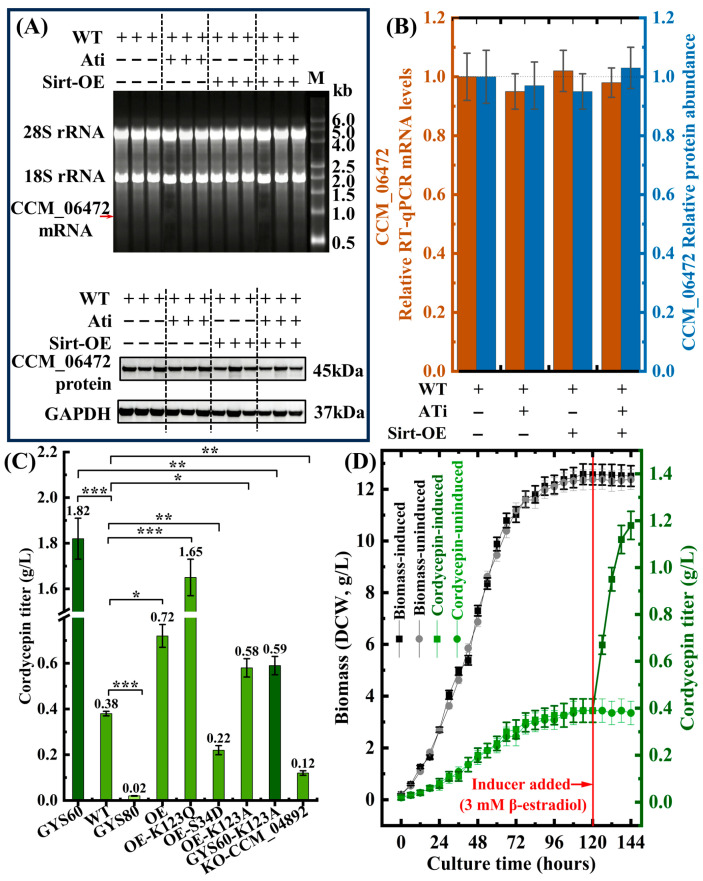
Functional validation of CCM_06472 as a post-translational regulatory node. (**A**) RNA electrophoresis and Western blot show unchanged transcription and translation. The red arrow indicates the position of the CCM_06472 mRNA transcript. (**B**) RT-qPCR and WB densitometry confirm unaltered mRNA and protein levels (all non-significants). (**C**) Acetylation-mimetic K123Q boosts cordycepin 4.3-fold; K123A reduces yield by 68%, * *p* < 0.05, ** *p* < 0.01, *** *p* < 0.001. (**D**) Inducible-K123Q increases cordycepin 3.1-fold without affecting biomass.

To confirm acetylation specificity, we inhibited CCM_06472 Lys123 acetylation using C646 treatment (reducing acetyltransferase activity by 78%) and Sirt2 overexpression (Figure 10D). ATi reduced Lys123 acetylation to 0.3-fold and cordycepin yield to 0.12 g/L, while Sirt-OE reduced acetylation to 0.1-fold and yield to 0.05 g/L. Combined treatment reduced acetylation to 0.08-fold and yield to 0.02 g/L. RT-qPCR and Western blot confirmed unchanged CCM_06472 expression across groups, eliminating transcriptional/translational effects as confounding factors (Figure 11A,B). These observations support Lys123 acetylation as a key regulatory determinant for CCM_06472 activity and cordycepin yield.

#### 3.4.5. PTM-Mimetic Mutations Confirm Rate-Limiting Role of CCM_06472

To validate that Lys123 acetylation of CCM_06472 is rate-limiting for cordycepin biosynthesis, we constructed PTM-mimetic/deficient strains (Figure 11C).

The acetylation-mimetic OE-CCM_06472-K123Q boosted cordycepin to 1.65 ± 0.08 g/L in WT (4.3-fold vs. WT, *p* < 0.01), reaching 91% of GYS60’s yield. Conversely, acetylation-deficient GYS60-K123A reduced yield by 68% (*p* < 0.01), supporting that Lys123 acetylation is necessary for high productivity. For industrial dynamic control, we engineered a β-estradiol-inducible CCM_06472-K123Q strain (Figure 11D). Induction at 120 h increased cordycepin 3.1-fold (to 1.18 ± 0.06 g/L at 144 h, *p* < 0.01) without altering biomass (~12.5 g/L DCW, *p* > 0.05). This orthogonal regulation decouples growth and production, an advance over traditional enzyme overexpression (only 1.9-fold yield gain).

### 3.5. Single-Node Engineering Overcomes Cordycepin Yield Limitations

To experimentally validate that the post-translational modification (PTM) state of CCM_06472 acts as the rate-limiting metabolic bottleneck governing cordycepin biosynthesis, we implemented minimal genetic engineering targeting this single regulatory node—avoiding the complexity of multi-gene pathway rewiring—and systematically characterized the production phenotypes of all engineered strains (Table 4). This approach tested whether manipulating CCM_06472’s PTM profile is sufficient to recapitulate or abrogate the high-yield trait of the natural high-producer strain GYS60.

Simple overexpression of CCM_06472 in wild-type (WT) achieved only a 1.9-fold yield enhancement (0.72 ± 0.05 g/L vs. 0.38 ± 0.01 g/L in WT, *p* < 0.05). However, introducing an acetylation-mimetic mutation (CCM_06472-K123Q) boosted cordycepin production to 1.65 ± 0.08 g/L, a 4.3-fold increase versus WT (*p* < 0.01) and 91% of GYS60’s yield (1.82 ± 0.09 g/L). This shows CCM_06472’s Lys123 acetylation-dependent activity limits cordycepin biosynthesis. Validation showed an acetylation-deficient mutation (CCM_06472-K123A) in GYS60 reduced cordycepin by 68% to 0.58 ± 0.04 g/L (*p* < 0.01). The phosphomimic mutation CCM_06472-S34D suppressed production to 0.22 ± 0.02 g/L, confirming an “acetylation-activation/phosphorylation-inhibition” mechanism. Knockout of CCM_04892 (malate dehydrogenase) reduced yield to 0.12 ± 0.01 g/L, showing acetyl-CoA supply enables CCM_06472 activation. We engineered an inducible CCM_06472-K123Q strain for dynamic control. Adding an inducer at 120 h post-inoculation increased cordycepin from 0.39 ± 0.02 g/L to 1.18 ± 0.06 g/L over 24 h, while biomass remained stable (12.51 ± 0.40 g/L), confirming orthogonal regulation.

### 3.6. A Proposed Acetyl-CoA-Gated Checkpoint Model

**Figure 12:**
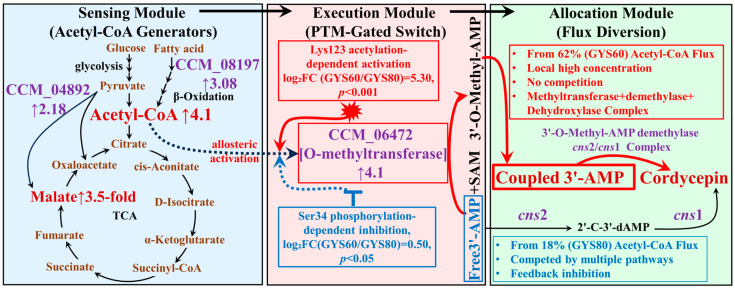
Model of the acetyl-CoA-gated metabolic checkpoint and cordycepin biosynthetic pathways in *C. militaris*. Note: Sensing module: Enhanced TCA cycle and β-oxidation drive 4.1-fold acetyl-CoA surplus. Execution module: Acetyl-CoA activates CCM_06472 via Lys123 acetylation (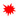) and inhibits it via Ser34 phosphorylation (⊣). Allocation module: High-yield GYS60 channels the majority of acetyl-CoA flux into cordycepin via a coupled assembly (no competition), while low-yield GYS80 only channels a small fraction (diverted by purine salvage and feedback inhibition). Dashed arrows indicate allosteric activation.

Integrating these findings, we propose an acetyl-CoA-gated checkpoint regulatory model that governs metabolic trade-offs in *C. militaris* cordycepin biosynthesis (Figure 12). The model operates through three core modules: (i) a sensing module (e.g., TCA cycle, β-oxidation) that generates an acetyl-CoA surplus; (ii) an execution module (PTM-gated enzymes like CCM_06472) where acetyl-CoA acts as a signal to activate key biosynthetic steps via acetylation; and (iii) an allocation module that ensures preferential channeling of flux into cordycepin biosynthesis.

In GYS60, this checkpoint is set to “high-fidelity” mode, efficiently converting abundant acetyl-CoA into cordycepin while minimizing diversion to competing pathways such as riboflavin synthesis. This mechanism explains why traditional enzyme overexpression often yields diminishing returns; it increases pipeline capacity without rewriting the cellular logic that governs flux allocation.

## 4. Discussion

### 4.1. Acetyl-CoA Signaling via CCM_06472 Gates Metabolic Flux to Cordycepin

Our integrative study reveals that exceptional cordycepin productivity in GYS60 is orchestrated by an acetyl-CoA-gated feedback loop, not merely by upregulating biosynthetic enzymes. This loop dynamically couples acetyl-CoA availability to the acetylation state of the key O-methyltransferase CCM_06472, effectively creating a ‘metabolic thermostat’ that implements precursor-directed regulation [26], prioritizing resource allocation to cordycepin biosynthesis when precursor supply is abundant (Figure 12).

A defining innovation in GYS60 is the adoption of a methylated intermediate branch (free 3′-AMP → 3′-O-Methyl-AMP → coupled 3′-AMP → cordycepin), which acts as a precision-engineered “metabolic sink.” Free 3′-AMP, a well-established crossroads molecule in the classical pathway [12,27], diverts only 18% of flux toward cordycepin in GYS80. By contrast, 3′-O-Methyl-AMP is chemically incompatible with these competing metabolic routes. This irreversibly locks 62% of acetyl-CoA-derived flux into cordycepin biosynthesis, achieving a 3.4-fold enhancement in flux fidelity.

This flux confinement is amplified by a unique “signal–flux coupling” mechanism. While classical pathway enzymes are insensitive to acetyl-CoA levels, CCM_06472’s activity correlates with acetyl-CoA, consistent with regulation via Lys123 acetylation in crude extract assays. This transforms the 4.1-fold higher acetyl-CoA surplus in GYS60 from a mere precursor pool into a regulatory signal, redirecting carbon away from wasteful byproducts (e.g., riboflavin) toward cordycepin.

CCM_06472 acts as a metabolic integrator linking precursor supply and cellular energetics, governed by an antagonistic acetylation–phosphorylation switch. Lys123 acetylation dominates under acetyl-CoA surplus and high ATP/NADH levels, activating cordycepin biosynthesis. In contrast, Ser34 phosphorylation, potentially mediated by fungal AMPK/Snf1-like energy-sensing kinases [28], prevails during nutrient limitation, conserving resources for primary metabolism. This design solves the precursor–product mismatch issue in traditional pathway engineering [29]. CCM_06472, a pathway-committing enzyme, only engages biosynthetic flux when metabolism permits [6]. This represents a more proactive feedforward activation strategy than simple feedback inhibition [7].

The structural dynamics of CCM_06472’s PTM sites follow established principles [23,24,25]. Molecular dynamics simulations show that WT CCM_06472 has low flexibility at Ser34 and moderate flexibility at Lys123, matching the 0.3–2.0 Å range ideal for functional PTM regulation. The acetylation-mimetic K123Q mutation increases Lys123 flexibility to 1.806 Å, favoring enzyme activation. Conversely, the phosphomimetic S34D mutation reduces Ser34 flexibility to 0.128 Å, consistent with phosphorylation-induced rigidification and inhibition. These conformational dynamics directly explain differential PTM recognition and establish a clear mechanistic link between protein structure, post-translational regulation, and cordycepin biosynthetic flux.

The methylated branch also addresses two longstanding bottlenecks in natural product biosynthesis. It circumvents product feedback inhibition [30]. We found that cordycepin accumulation inhibits cns1 in the classical pathway, but 3′-O-Methyl-AMP avoids this repression. Also, it prevents intermediate diversion through spatial coupling. The demethylation of 3′-O-Methyl-AMP is kinetically coupled to cns1/cns2, sequestering released 3′-AMP in a metabolic microcomplex. This generates a locally high concentration of coupled 3′-AMP, which outcompetes non-target enzymes and displaces cordycepin from cns1’s active site, relieving inhibition.

GYS60’s high-yield phenotype is driven by a coherent regulatory architecture. The acetyl-CoA checkpoint, via a PTM-gated methyltransferase, channels flux into a dedicated, insulated biosynthetic branch. The stark contrast between the limited success of traditional enzyme overexpression (OE-CCM_06472, 1.9-fold boost) and the dramatic effect of rewiring its PTM logic underscores that reprogramming cellular decision-making, rather than merely expanding pathway capacity, is key to breaking yield ceilings.

### 4.2. A Conserved Acetylation–Phosphorylation Switch in Fungal Specialized Metabolism

The acetyl-CoA-mediated checkpoint we describe in *C. militaris* may represent a testable hypothesis for conserved regulatory paradigms in fungal specialized metabolism. We propose three testable hypotheses for broader applicability: (i) key committing enzymes in fungal specialized metabolic pathways may function as PTM-gated nodes; (ii) acetylation (or other precursor-derived PTMs) may couple precursor abundance to pathway activation; and (iii) such PTM-gated flux allocation may enhance metabolic fidelity by minimizing intermediate diversion.

Notably, parallel PTM-mediated regulatory patterns have been observed in other fungal secondary metabolic pathways, including aflatoxin biosynthesis in *Aspergillus flavus* [31,32], diterpenoid production in *Fusarium fujikuroi* [33], and penicillin biosynthesis in *Penicillium chrysogenum* [5,34]. These examples align with the core logic of the acetyl-CoA-gated checkpoint we describe in *C. militaris*, suggesting that this regulatory strategy represents a testable hypothesis for broader conservation across fungal specialized metabolism. Direct validation of this hypothesis in diverse systems represents a key avenue for future investigation.

Our work in *C. militaris* provides the most mechanistic dissection of this logic to date, with structural dynamics data aligning with broader PTM-flexibility principles [22,23,24]. The antagonistic acetylation–phosphorylation regulation at CCM_06472 (Figure 9A) exemplifies how fungi can employ PTM-based logic gates to integrate multiple signals, such as precursor abundance (via acetyl-CoA) and potentially cellular energy status, to enable precise, dynamic control of metabolic commitment [7]. Such combinatorial PTM codes, functioning as binary or multi-state switches, are a well-documented mechanism for precise control of protein function in eukaryotes [35], offering an elegant solution to the universal challenge of balancing growth with production.

### 4.3. PTM-Proteomics Enables Causal Discovery of Metabolic Regulators

Methodologically, our study demonstrates the power of comparative PTM-proteomics to move beyond correlative omics. While transcriptomics often poorly predicts metabolic flux due to post-transcriptional regulation [36,37], profiling of PTMs in isogenic strains with divergent phenotypes allowed us to establish causality at the functional protein level. We used it to pinpoint CCM_06472 Lys123 acetylation as key, with a strong activity-acetylation correlation (*r* = 0.92) validating the method.

This “proteome-first” approach provides a general blueprint for deciphering empirically obtained, high-performing strains. The success of our subsequent minimal engineering strategy, where a single PTM-mimetic mutation recapitulated most of the high-yield phenotype, validates the precision and efficiency of this methodology. Future efforts can systematically employ comparative PTM profiling to uncover hidden regulatory adaptations, accelerating the translation of phenotypic advantages into rational design principles.

### 4.4. Single PTM Mutation Enables Rational Rewiring of Fungal Cell Factories

Recently, metabolic checkpoint engineering, represented by post-translational modification (PTM)-mediated switch regulation, has become a new frontier in synthetic biology [9]. This paradigm emphasizes targeting regulatory nodes rather than simply modifying pathways, which is highly consistent with our strategy of achieving a 4.3-fold yield increase through a single point mutation (K123Q), providing a new path for the rational design of fungal cell factories.

Our findings align with several feasible strategies to advance metabolic checkpoint engineering, including rational design of PTM sites for orthogonal enzyme variants [38], development of synthetic metabolite sensors [39], and the dynamic control of modification states using optogenetic or chemical tools [40]. Our work validates that the K123Q acetylation-mimetic mutation (4.3-fold yield boost) exemplifies PTM site engineering [38]. Additionally, β-estradiol-inducible CCM_06472-K123Q strains (Figure 11D) confirm robust chemical-based dynamic control [40].

The stark contrast between the limited success of traditional enzyme overexpression (OE-CCM_06472, 1.9-fold boost) and the dramatic effect of rewiring its PTM logic underscores that reprogramming cellular decision-making, rather than merely expanding pathway capacity, is key to breaking yield ceilings.

### 4.5. Generalizable Checkpoint Principle for Microbial Metabolism

Our study reframes the paradigm for yield improvement by moving beyond static pathway optimization to decipher the dynamic regulatory logic cells use to allocate resources. The discovery of the acetyl-CoA checkpoint not only provides a mechanistic solution for cordycepin overproduction, but also establishes a system-specific regulatory principle for metabolic trade-off engineering in *C. militaris*. To translate this principle from checkpoint discovery into checkpoint engineering, key frontiers must be navigated. The immediate task is to fully elucidate the molecular apparatus, the specific acetyltransferase and kinase, that constitute the CCM_06472 switch. As a testable hypothesis for future work, this acetyl-CoA-gated logic may be explored by engineering analogous PTM-sensing modules in other fungal systems. Success across diverse systems would transform it from a compelling case study into a universal design rule for synthetic biology. Ultimately, by integrating such precise regulatory control with genome-scale models, we can learn to speak the cell’s own metabolic language, enabling the predictive rewiring of microbial factories for optimal performance.

## 5. Conclusions

This study elucidates the molecular basis of high cordycepin production in *Cordyceps militaris*, revealing a novel paradigm wherein high yield is governed by an acetyl-CoA-gated metabolic checkpoint rather than overexpression of biosynthetic enzymes. Central to this mechanism is the O-methyltransferase CCM_06472, which acts as a PTM-gated molecular switch. Its activity is modulated by Lys123 acetylation and Ser34 phosphorylation, consistent with a model linking intracellular acetyl-CoA availability to metabolic flux toward cordycepin. This dual role of acetyl-CoA as both precursor and signaling molecule creates an efficient, self-reinforcing circuit for metabolic resource allocation.

Our work demonstrates methodological and biological understanding. Methodologically, comparative PTM-proteomics proves effective for moving beyond correlative omics to uncover causal, post-translational regulators of industrial phenotypes. Biologically, the acetyl-CoA checkpoint establishes a novel regulatory logic for growth–production trade-offs. Given the centrality of acetyl-CoA, this principle represents a testable hypothesis for extension to diverse fungal and microbial secondary metabolisms. Critically, we validated this mechanism by engineering a single PTM-mimetic mutation that largely recapitulates the high-yield phenotype, establishing CCM_06472 as a robust target for rational strain improvement.

Consequently, this work reorients metabolic engineering from static pathway optimization to the dynamic reprogramming of cellular regulatory networks. The checkpoint engineering framework provides a blueprint for designing next-generation cell factories with intelligent resource allocation. By hijacking the cell’s inherent metabolic decision-making logic, we pave the way for precisely overcoming longstanding yield ceilings in microbial biosynthesis.

## Figures and Tables

**Table 1:** Protein identification and quantification statistics.

Strain	Total Proteins	High-Confidence Proteins	Average Coverage (%)	Median Coverage (%)	Pearson Correlation
Wild-type	2356 ± 28	1980 ± 27	25 ± 1	18 ± 1	0.92 ± 0.01
GYS60	2410 ± 32	2003 ± 15	24 ± 1	17 ± 1	0.91 ± 0.01
GYS80	2378 ± 25	1985 ± 15	26 ± 1	19 ± 1	0.92 ± 0.01

**Table 2:** Key PTM site analysis.

Protein ID	Functional Annotation	Modification Site	Modification Type	log_2_ (Fold Change) (GYS60/GYS80)	*p*-Value
CCM_06472	O-methyltransferase	Lys123	Acetylation	5.30 ± 0.40	<0.001
CCM_06472	O-methyltransferase	Ser34	Phosphorylation	0.50 ± 0.10	<0.05
CCM_04892	Malate dehydrogenase	Lys207	Acetylation	1.94 ± 0.23	<0.05
CCM_01234	Pyruvate kinase	Ser158	Phosphorylation	2.10 ± 0.30	<0.01
CCM_05678	Isocitrate dehydrogenase	Lys315	Acetylation	1.80 ± 0.20	<0.05
CCM_09101	Glutathione peroxidase	Ser45	Phosphorylation	0.60 ± 0.10	<0.05

**Table 3:** Differentially expressed proteins (representative subset).

Gene Name	Molecular Function	log_2_ (Fold Change)		
		GYS60 vs. WT	GYS80 vs. WT	GYS60 vs. GYS80
*CCM_06472*	O-methyltransferase	1.56 ± 0.42 ↑ *	−0.48 ± 0.13 ↓ *	5.31 ± 0.38 ↑ **
*CCM_04892*	Malate dehydrogenase	2.18 ± 0.63 ↑ *	1.53 ± 0.40	3.69 ± 0.19 ↑ **
*CCM_08197*	Acyl-CoA dehydrogenase	3.08 ± 1.38 ↑ *	−0.97 ± 1.64	3.58 ± 1.99 ↑ **
*CCM_03688*	Indoleamine 2,3-dioxygenase	2.12 ± 0.71 ↑ *	1.80 ± 0.57	3.30 ± 1.37 ↑ **
*CCM_04591*	Serine protein kinase	−1.68 ± 1.89 ↓ *	−0.48 ± 0.45	0.23 ± 0.49

Note: * *p* < 0.05, ** *p* < 0.01. ↑: protein abundance increased; ↓: protein abundance decreased.

**Table 4:** Cordycepin titers in engineered strains.

Strain	Genotype	Cordycepin Titer (g/L)	Fold Change Relative to WT	Statistical Significance
Wild-type	WT	0.38 ± 0.01	1.0×	Reference
GYS60	High-yield mutant	1.82 ± 0.09	4.8×	*p* < 0.01
OE-CCM_06472	WT overexpression	0.72 ± 0.05	1.9×	*p* < 0.05
OE-CCM_06472-K123Q	Acetylation-mimetic	1.65 ± 0.08	4.3×	*p* < 0.01
OE-CCM_06472-S34D	Phosphorylation-mimetic	0.22 ± 0.02	0.6×	*p* < 0.01
OE-CCM_06472-K123A	Acetylation-deficient	0.58 ± 0.04	1.5×	*p* < 0.05
KO-CCM_04892	Malate dehydrogenase knockdown	0.12 ± 0.01	0.32×	*p* < 0.01
Inducible K123Q	Chemically inducible	1.18 ± 0.06 (induced)	3.1×	*p* < 0.01
Inducible K123Q	Chemically inducible	0.39 ± 0.02 (uninduced)	1.0×	NS

Notes: NS: not significant.

## Data Availability

The original contributions presented in this study are included in the article. All raw data generated in this study have been deposited in the iPROX public repository (https://www.iprox.cn/, accessed on 18 March 2026, accession number: IPX0016387000) and MetaboLights (https://www.ebi.ac.uk/metabolights/ (accessed on 18 March 2026), accession number: REQ20260326218169). Further inquiries can be directed to the corresponding authors.

## Abbreviations

The following abbreviations are used in this manuscript:

3′-AMP	3′-Adenosine monophosphate
3′-O-Methyl-AMP	3′-O-methyl-adenosine monophosphate
ACS	Acetyl-CoA synthetase
ACN	Acetonitrile
ATi	Acetylation inhibitor
BCA	Bicinchoninic acid
CV	Coefficient of variation
CCM_06472	O-Methyltransferase
CCM_04892	Malate dehydrogenase
Cns1	Cordycepin synthetase 1, CCM_04436, dehydrogenases
Cns2	Cordycepin synthetase 2, CCM_04437, phosphohydrolases
Cns3	Cordycepin synthetase 3, CCM_04438, ATP phosphoribosyltransferases
DAMs	Differentially abundant metabolites
DCW	Dry cell weight
DDA	Data-dependent acquisition
DEPs	Differentially expressed proteins
ECL	Enhanced Chemiluminescence
EC_50_	Half-maximal effective concentration
ESI+	Electrospray ionization positive
FASP	Filter-aided sample preparation
FDR	False discovery rate
FL	Fractional labeling
GO	Gene Ontology
GYS60	High-yield cordycepin strain
GYS80	Low-yield cordycepin strain
HCD	Higher-energy collisional dissociation
HPLC-UV	High-performance liquid chromatography-ultraviolet detection
HO	High overexpression
IP-MS/MS	Immunoprecipitation–tandem mass spectrometry
KEGG	Kyoto encyclopedia of genes and genomes
KO	Knockout
LC-MS/MS	Liquid chromatography-tandem mass spectrometry
NCE	Normalized collision energy
OE	Overexpression
PCA	Principal component analysis
PTM	Post-translational modification
PVDF	Polyvinylidene difluoride
Q-TOF	Quadrupole time-of-flight
RMSF	Root Mean Square Fluctuation
RT-qPCR	Reverse transcription–quantitative polymerase chain reaction
SAM	S-Adenosylmethionine
Sirt-OE	Sirtuin overexpression
SDS-PAGE	Sodium dodecyl sulfate-polyacrylamide gel electrophoresis
SRM	Selected reaction monitoring
TCA	Tricarboxylic acid cycle
TEAB	Triethylammonium bicarbonate
TMT	Tandem mass tag
UPLC-MS/MS	Ultra-performance liquid chromatography–tandem mass spectrometry
Vmax	Maximum reaction velocity
WB	Western blot
WT	Wild-type
